# Corrosion Behavior of Titanium in Artificial Saliva by Lactic Acid

**DOI:** 10.3390/ma7085528

**Published:** 2014-07-28

**Authors:** Qing Qu, Lei Wang, Yajun Chen, Lei Li, Yue He, Zhongtao Ding

**Affiliations:** 1 School of Chemical Science and Technology, Yunnan University, Kunming 650091, China; E-Mails: 12012001133@mail.ynu.edu.cn (L.W.); yajunchenqulee@gmail.com (Y.C.); 12013001123@mail.ynu.edu.cn (Y.H.); 961779457lee@gmail.com (Z.D.); 2 Laboratory for Conservation and Utilization of Bio-Resources, Yunnan University, Kunming 650091, China; E-Mail: leelei@ynu.edu.cn

**Keywords:** corrosion, titanium, artificial saliva, lactic acid, corrosion mechanism

## Abstract

As one of the main products produced by oral microorganisms, the role of lactic acid in the corrosion of titanium is very important. In this study, the corrosion behavior of titanium in artificial saliva with and without lactic acid were investigated by open-circuit potentials (OCPs), polarization curves and electrochemical impedance spectroscopy (EIS). OCP firstly increased with the amount of lactic acid from 0 to 3.2 g/L and then tended to decrease from 3.2 to 5.0 g/L. The corrosion of titanium was distinctly affected by lactic acid, and the corrosion rate increased with increasing the amount of lactic acid. At each concentration of lactic acid, the corrosion rate clearly increased with increasing the immersing time. Results of scanning electron microscopy (SEM) also indicated that lactic acid accelerated the pitting corrosion in artificial saliva. A probable mechanism was also proposed to explain the experimental results.

## 1. Introduction

Since titanium is considered to have high biological affinity, it has been intensively applied in the manufacturing of biomedical devices since the 1980s [1]. In the past few decades, the increasing interest in using titanium in dentistry for bridges and crowns, metal-ceramic restorations, is due to its excellent properties of biocompatibility, low density, low thermal conductibility, mechanical behavior and corrosion resistant [2,3,4,5,6,7,8]. The good corrosion resistance of titanium is the result of the presence of a protective and self-adherent oxide film of a thickness of 2–6 nm formed on the titanium surface, which is mainly composed of titanium dioxide (TiO_2_) [9,10,11,12,13,14].

However, titanium and its alloys are often damaged during their use in some complicated environments, such as oral environments. There also have been several studies describing patients who did not adapt to titanium [15,16] or patients allergic to titanium [17,18,19,20], which is an important issue to resolve [21,22]. To find out the real reason, one of the key issues at present is to learn the corrosion behavior and mechanism of titanium in oral environments. In fact, the elements that cause the corrosion of titanium in oral environments are quite a lot, but in general, microbially-influenced corrosion (MIC) is deemed to be the main reason. Just like MIC in other environments, the mechanisms involved in oral environments are very complicated, as the process is affected by many factors, but the common viewpoint involved is that the metabolic product has a great impact on MIC. As one of the main metabolic substances in oral environments, lactic acid exists naturally in the human oral cavity as a product of the oral cell tissues and of the bacteria [23]. Thus, the role of lactic acid in the corrosion of titanium is rather important to further explore the effect of MIC in oral environments. There also are several brief studies concerning the effect of lactic acid on titanium in oral environments. Mabilleau *et al.* [24] investigated the corrosion resistance of CP-Ti disks for nine days of immersion in different test solutions based on artificial saliva containing F^‑^, H_2_O_2_ and lactic acid by AFM and SEM. Their results showed that the surface roughness was highly increased when titanium disks were immersed in artificial saliva containing F^−^, H_2_O_2_ and lactic acid. Koike and Fujii [25,26] reported that titanium has a high resistance to corrosion in physiological saline and artificial saliva by immersion tests and that it was dissolved and became discolored when in contact with lactic acid or formic acid. Takahashi *et al.* [27] evaluated the corrosion resistance of Ti-Ag alloys in a 1% lactic acid solution. They showed that the Ti-Ag alloys had excellent corrosion resistance that was comparable or superior to that of pure titanium. Koike and Fujii [28] studied the corrosive properties of titanium in 128 mmol/L of lactic and formic acids at pH 1.0–8.5 for three weeks at 37 °C. They declared that the corrosive properties of titanium were markedly dependent on pH in formic acid and relatively less dependent on pH in lactic acid. However, up to now, there have been few reports studying the effect of lactic acid amount, and the mechanism remains unclear.

Therefore, in order to provide an in-depth understanding of the corrosion behavior of titanium in complex physiological solutions for clinical use, especially under various conditions in the mouth, and to offer significant guidance for clinical applications of titanium and its alloys, it is of importance to identify the mechanism of titanium corrosion in saliva containing lactic acid. The purpose of this work is to study the corrosion behavior of titanium and to explore the probable mechanism in artificial saliva containing lactic acid.

## 2. Results and Discussion

### 2.1. pH Results

**Figure 1:**
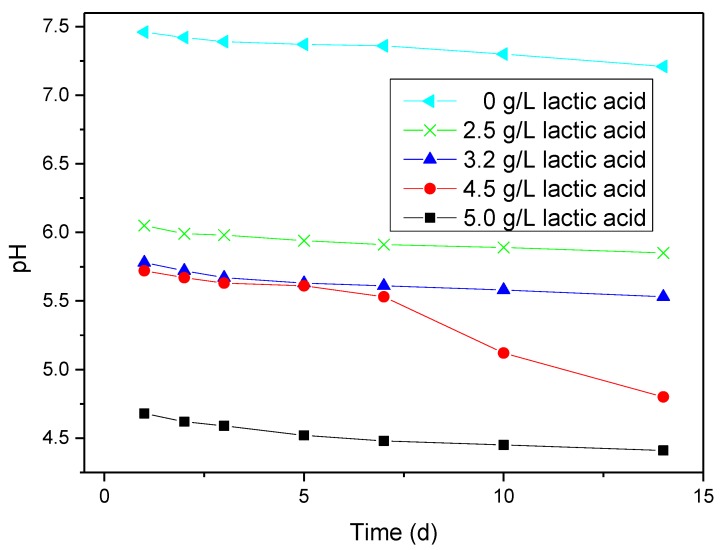
pH values of artificial saliva containing a working electrode with and without lactic acid for different days.

Figure 1 gives the pH values of artificial saliva containing a working electrode with and without lactic acid for different days. As can be seen from this figure, pH clearly decreases with increasing the amount of lactic acid at each time, which, in turn, suggests the weak acidity of lactic acid. Moreover, the pH slightly decreases with the increase in immersion time at the same amount of lactic acid. This also indicates that the dissolution of titanium in artificial saliva solution with and without lactic acid tends to decrease the pH value.

### 2.2. Open-Circuit Potential (OCP) Measurements

**Figure 2:**
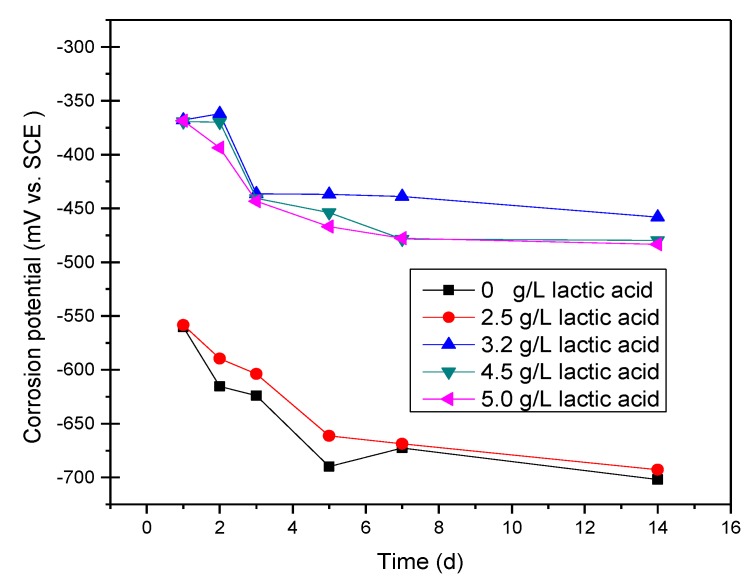
Open-circuit potential of titanium immersed in artificial saliva containing different amounts of lactic acid. SCE, saturated calomel electrode.

Figure 2 presents the open-circuit potential (OCP) of titanium after immersion in artificial saliva with and without lactic acid solutions during different times. It is clear that OCP decreases quickly with the increase time in the initial days and then tends to be stable. Additionally, OCP increases with increasing the amount of lactic acid from 0 to 3.2 g/L, but slightly decreases from 3.2 to 5.0 g/L. This implies that the amount of lactic acid significantly affects the dissolution of titanium. With increasing the amount of lactic acid from 0 to 3.2 g/L, the adsorption of lactic acid on the surface increases, which results in an increase of OCP; then, the adsorption may be saturated when the concentration is higher or equal to 3.2 g/L, that is the detachment becomes more important, which causes a slight decrease of OCP.

### 2.3. Electrochemical Impedance Spectroscopy (EIS) Studies

**Figure 3:**
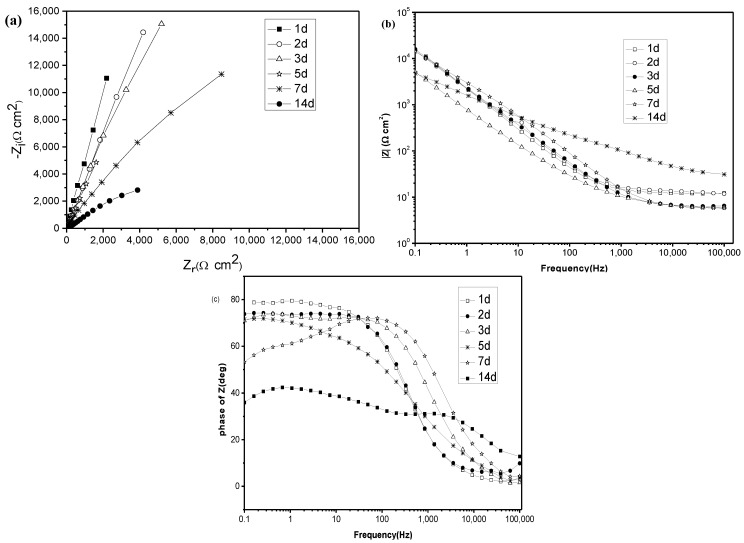
EIS of titanium immersed for different times in artificial saliva without lactic acid. (**a**) Nyquist plots; (**b**) bode modulus diagrams; and (**c**) bode phase angle diagrams.

**Figure 4:**
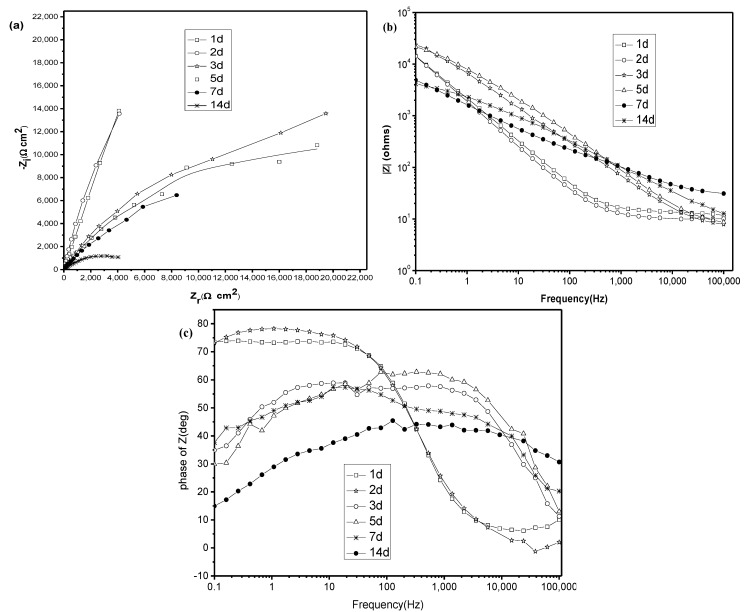
EIS of titanium immersed for different times in artificial saliva containing 5.0 g/L lactic acid. (**a**) Nyquist plots; (**b**) bode modulus diagrams; and (**c**) bode phase angle diagrams.

Figure 3 and Figure 4 show the Nyquist plots of titanium immersed in artificial saliva without and with 5.0 g/L lactic acid. Nyquist plots are respectively presented in Figure 3a and Figure 4a. The corresponding Bode modulus diagrams and Bode phase angle diagrams are also presented in Figure 3b,c and Figure 4b,c. The EIS in the presence of other amounts of lactic acid are the same as those in the presence of 5.0 g/L lactic acid and also not presented here.

**Figure 5:**
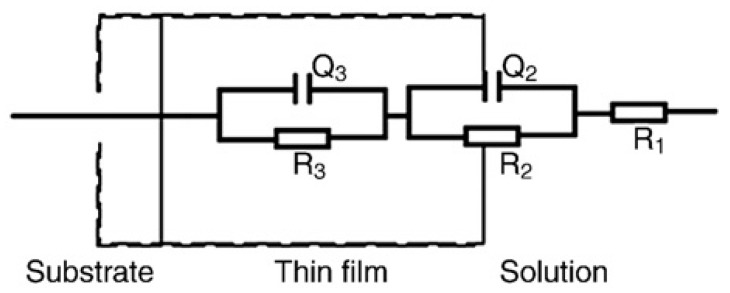
Equivalent circuit.

From Figure 3a and Figure 4a, it is clearly found that all of the diagrams are a part of the imperfect semicircles, and this is attributed to the frequency dispersion [29,30,31]. The fact that impedance diagrams have a depressed semicircular appearance shows that the corrosion of titanium is mainly controlled by a charge transfer process, and the presence of lactic acid does not change the mechanism of dissolution. In the artificial saliva medium with and without lactic acid, the diagrams show larger values of impedance. The larger values of impedance suggest the existence of a passive film with and without lactic acid [30], but the film becomes more non-protective, because the values of impedance become smaller with increasing immersion time [31]. The same phenomenon could be found in Bode modulus diagrams (Figure 3b and Figure 4b). Furthermore, Bode phase angle diagrams obtained for titanium electrodes (Figure 3c and Figure 4c) clearly show a two-time constant behavior. According to Mansfeld [31] and Tian *et al.* [32], two-time constants relate to a two-layer structure developed during the corrosion. Thus, a two-time constant equivalent circuit, as shown in Figure 5, can be used for pure electronic models that could verify or rule out mechanistic models and enable the calculation of numerical values corresponding to the electrochemical system under investigation [33]. 

In the equivalent circuit, R_1_ is the solution resistance; R_2_ is the charge transfer resistance; and R_3_ is the film resistance. Q_2_ and Q_3_ are the constant phase elements (CPEs) to replace the double-layer capacitance at the film-solution interface and the capacitance of the film, respectively. The constant phase element (CPE) is usually used instead of a capacitance to account for the non-ideal capacitance response, due to the almost complete absence of pure capacitance in the real electrochemical process. *n* is a mathematical expression where 0 ≤ *n* ≤ 1. If *n* = 0, the impedance is entirely resistance, while it is capacitance if *n* = 1. Additionally, 0 < *n* < 1 represents one of the various causes for deviation from the ideal capacitance, which is related to the surface roughness, adsorption film, porous layer formation, and so on [29]. It is quite well-known that surface roughness is of considerable importance in creating pits, which facilitate pitting corrosion [30]. The EIS parameters are fitted by this model and given in Table 1. As can be seen from this table, at the same amount of lactic acid, with increasing the immersion time, the transfer resistance (R_2_) obviously decreases, indicating that the corrosion is accelerated. The addition of lactic acid to solution displayed a decrease of R_2_ values, suggesting that lactic acid accelerates the corrosion of titanium. The solution resistance (R_1_) decreased with immersion time increase, because titanium released metal ions after being corroded, causing the solution resistance diminution and enhancing electrical conductivity. For the film resistance (R_3_), it has a smaller value compared to R_2_ but does not have a clear trend, due to its variation in magnitude with time for the formation and desorption of the film-electrolyte interface; generally speaking, R_3_ decreases with the addition of lactic acid, suggesting that the resistance of the film formed on the titanium surface to lactic acid is rather lower. The values of *n*_1_, *n*_2_ decrease with the increase in the immersion time, which further supports the idea that surface inhomogeneity is increasing due to the pitting of titanium. Additionally, an enhanced tendency of the capacitance of the film (Q_3_) also can be found from Table 1 with the increase of time, showing that the corrosion resistance of the film decreases with the increase of time.

**Figure 6:**
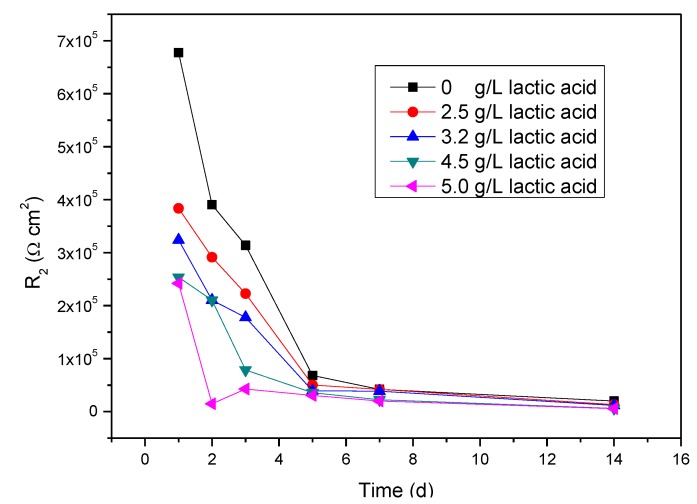
R_2_ of titanium immersed in artificial saliva containing different amounts of lactic acid.

To more directly demonstrate the effect of lactic acid on the corrosion of titanium, Figure 6 further presents the relationship between R_2_ and immersion time in artificial saliva containing different amounts of lactic acid. From this figure, it is clear that R_2_ decreases with increasing of the immersion time at each amount of lactic acid. Furthermore, R_2_ also decreases with the increase of the amount of lactic acid at the same time; namely the corrosion of titanium is noticeably accelerated by lactic acid, and the corrosion increases with the increase in the amount of lactic acid.

**Table 1:** EIS parameters of titanium immersed for different times in artificial saliva containing different amounts of lactic acid.

Lactic acid	Time	R_1_	Q_2_	n_1_	R_3_	Q_3_	n_2_	R_2_
g/L	days	Ω·cm^2^	Ω^−^^1^·cm^−2^·s^n^		Ω·cm^2^	Ω^−^^1^·cm^−2^·s^n^		Ω·cm^2^
0.0	1	17.45	360.5	1.000	98.14	97.96	1.000	6.776 × 10^5^
	2	13.18	224.9	0.8957	150.9	96.00	0.8950	3.904 × 10^5^
	3	6.328	390.7	0.8525	444.1	91.99	0.8889	3.139 × 10^5^
	5	5.841	205.6	0.8342	653.1	303.3	0.8714	6.829 × 10^4^
	7	5.741	124.8	0.7561	980.3	920.4	0.8191	4.209 × 10^4^
	14	5.370	229.3	0.7335	988.2	803.3	0.6076	1.999 × 10^4^
2.5	1	10.01	923.5	0.9906	6.283	91.42	0.9216	3.837 × 10^5^
	2	9.681	131.7	0.8798	8.922	85.96	0.8169	2.914 × 10^5^
	3	8.423	397.1	0.8458	10.53	104.1	0.8316	2.227 × 10^5^
	5	7.605	381.6	0.8359	30.83	956.0	0.8012	5.030 × 10^4^
	7	6.794	507.2	0.8123	61.64	872.3	0.7169	4.1998 × 10^4^
	14	5.025	773.6	0.7737	8.940	796.2	0.6574	1.336 × 10^4^
3.2	1	6.896	928.8	0.9959	7.392	94.92	0.8924	3.240 × 10^5^
	2	6.021	566.3	0.9378	8.028	20.16	0.8854	2.103 × 10^5^
	3	5.106	793.8	0.8991	1487	15.67	0.8871	1.779 × 10^5^
	5	4.705	96.5	0.8086	973.2	11.63	0.8655	3.944 × 10^4^
	7	3.182	62.3	0.5844	424.7	946.8	0.7378	3.877 × 10^4^
	14	2.737	134.7	0.5987	366.3	134.0	0.6570	1.172 × 10^4^
4.5	1	6.875	152.3	0.9129	9.001	98.98	0.8812	2.538 × 10^5^
	2	6.001	55.63	0.9108	8.128	22.16	0.8354	1.903 × 10^5^
	3	5.549	125.7	0.8694	4.900	19.05	0.7465	7.584 × 10^4^
	5	5.738	33.75	0.8648	48.89	96.72	0.7126	3.574 × 10^4^
	7	4.922	50.97	0.8040	11.19	944.5	0.6515	2.253 × 10^4^
	14	3.901	129.0	0.5053	121.6	182.3	0.6427	6,124
5.0	1	5.191	281.5	0.8100	8.315	98.34	0.8390	2.421 × 10^4^
	2	4.240	882.8	0.7788	10.13	40.44	0.8770	1.483 × 10^4^
	3	3.280	7.823	0.7588	5.109	38.48	0.7883	4.274 × 10^4^
	5	2.980	44.64	0.7395	1546	34.10	0.7279	3.054 × 10^4^
	7	2.380	224.2	0.5377	90.03	790.1	0.6072	1.998 × 10^4^
	14	2.481	81.19	0.5219	45.68	117.9	0.5094	5,726

### 2.4. Polarization Curves Tests

**Figure 7:**
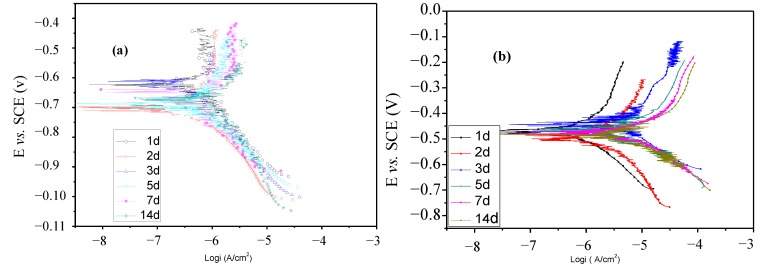
Polarization curves of titanium immersed in artificial saliva without (**a**) and with 5.0 g/L lactic acid (**b**) for different immersion times.

To elucidate the role of lactic acid in the corrosion of pure titanium more clearly, polarization curves of titanium immersed in artificial saliva without and with 5.0 g/L lactic acid for different immersion times were respectively recorded and plotted in Figure 7a,b. The polarization curves in the presence of other amounts of lactic acid are the same as those in the presence of 5.0 g/L lactic acid and also not presented here. As can be seen from these figures, in the absence of lactic acid, only slight shifts in the anodic and cathodic branches are observed with the immersion time increase, but the anodic current densities remain unchanged with the increase of the applied potential before seven days of immersion, that is the passivation characteristic can be found before seven days of immersion; In the presence of 5.0 g/L lactic acid, the anodic and cathodic current densities increase obviously with the increase in the immersion time compared to that in the absence of lactic acid, suggesting that lactic acid can accelerate the corrosion of titanium, and the corrosion increases with increasing of the immersion time.

Polarization parameters, including the corrosion potential (*E*_corr_), cathodic Tafel slopes (β_c_), anodic Tafel slopes (β_a_) and corrosion current density (*I*_corr_) obtained by extrapolation of the curves, are shown in Table 2. From Table 2, we can observe that, similar to the results from OCP, the corrosion potential decreases with the increase time in the initial days and then tends to be stable, but the corrosion potential obviously increases when lactic acid is added to artificial saliva; as for *I*_corr_, at the same amount of lactic acid, *I*_corr_ gradually increases with the increase of immersion duration in the artificial saliva medium; furthermore, at the same immersion time, *I*_corr_ increases with the increasing amount of lactic acid, indicating that the corrosion is aggravated. These results are in good agreement with the results obtained from EIS.

**Table 2:** Polarization parameters of titanium in artificial saliva containing different amounts of lactic acid.

Lactic acid g/L	Time d	E_corr_ mV	I_corr_ μA/cm^2^	b_a_ mV/dec	b_c_ mV/dec
0.0	1	−560.452	0.221	166.573	194.813
	2	−615.427	0.464	156.569	523.672
	3	−623.942	0.664	247.394	279.867
	5	−689.747	0.727	188.126	460.020
	7	−672.599	1.367	325.928	247.620
	14	−701.093	1.597	323.461	167.816
2.5	1	−558.415	0.483	243.754	832.327
	2	−589.474	0.727	188.126	460.020
	3	−603.838	0.856	222.604	480.795
	5	−661.228	1.270	257.742	752.736
	7	−688.779	1.957	256.943	127.068
	14	−692.844	2.957	112.101	390.369
3.2	1	−367.863	0.605	113.954	193.862
	2	−362.064	0.940	145.262	181.065
	3	−436.498	1.185	177.877	154.509
	5	−437.044	1.718	156.346	298.723
	7	−438.890	2.374	98.356	221.833
	14	−458.169	3.602	119.909	254.598
4.5	1	−309.275	1.158	317.385	331.341
	2	−369.804	1.959	285.187	620.453
	3	−440.754	2.587	204.845	337.687
	5	−433.871	3.216	117.898	250.677
	7	−478.407	4.373	129.221	225.129
	14	−479.760	5.003	150.240	193.490
5.0	1	−368.589	2.226	310.458	760.726
	2	−393.751	3.420	304.961	405.529
	3	−443.304	3.979	134.820	276.343
	5	−466.912	4.680	143.859	226.051
	7	−477.709	5.615	136.394	233.153
	14	−483.366	7.788	160.194	236.892

### 2.5. Scanning Electron Microscopy Analysis

**Figure 8:**
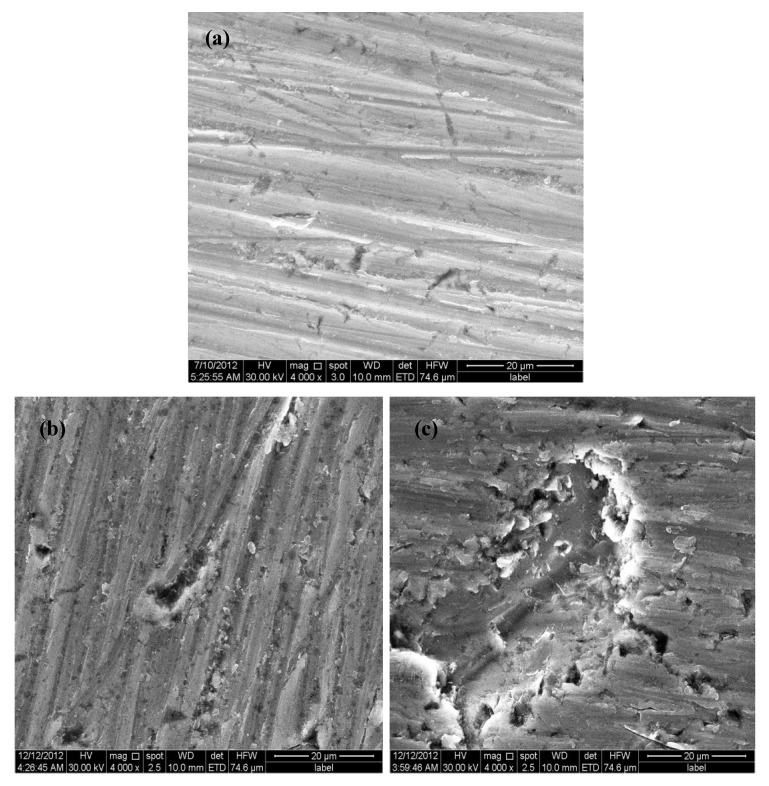
Surface morphologies of titanium immersed in artificial saliva: (**a**) in the absence of lactic acid; and (**b**) in the presence of 3.2 g/L; and (**c**) 5.0 g/L lactic acid for 14 days.

In order to further study the surface morphology of titanium after immersion, scanning electron microscopy is used to characterize the corrosion surface. Figure 8a–c show the surface morphologies of titanium immersed in artificial saliva in the absence of lactic acid and in the presence of 3.2 and 5.0 g/L lactic acid for 14 days, respectively. As can be seen from these figures, the surface in the absence of lactic acid looks rather smoother, that is the corrosion attack in the absence of lactic acid is more uniform in character, and there is little tendency towards local enrichment of products on the surface. In addition, parallel features, which can be associated with abrading scratches, are also observed in Figure 8a. However, in the presence of lactic acid, pitting corrosion can be distinctly observed; by comparison, the pits in Figure 8c are obviously larger and deeper than those in Figure 8b. Therefore, the addition of lactic acid can significantly affect the formation of pitting. Consequently, at the microscopic level, the effect of lactic acid on generating pitting cannot be ignored. Additionally, the adsorption of lactic acid in the micro-point of pitting corrosion can hinder titanium passivation, providing a necessary premise and driving force for generating macro-pitting corrosion.

### 2.6. Corrosion Mechanism

As it is well known that the good corrosion resistance of titanium is the result of the presence of a protective and self-adherent oxide film of a thickness of 2–6 nm formed on the titanium surface, which is mainly composed of titanium dioxide (TiO_2_) [16,19,20,21,22,23], however TiO_2_ still has a weaker dissolving capacity in aqueous solution, due to the following dissolution equilibrium [20,21]:

TiO_2_ ↔ TiO^2+^ + O^2−^ (1)

However, TiO^2+^ is unstable and easy to hydrolyze in solution containing chloride ions [34],

TiO^2+^ + Cl^−^ + 2H_2_O ↔ [Ti(OH)_3_]·Cl + H^+^ (2)

The hydrolysis will decrease the pH to a certain extent, that is the corrosion of titanium in artificial saliva solution will cause the pH decrease, as can be seen from the pH variation in the corrosion process of titanium (Figure 1). Figure 1 shows that pH value slightly decreases with the increase of the immersion time in artificial saliva without lactic acid. This experimental result, in turn, suggests the possibility of the above-mentioned mechanism.

As a weak electrolyte, lactic acid will ionize to lactate (L^−^) and H^+^ ions when lactic acid is added to solution. On the one hand, the ionization of lactic acid will decrease the pH value (Figure 1); thus, the pH value in the presence of lactic acid is lower than that in the absence of lactic. On the other hand, the anion, lactate (L^−^), ionized by lactic acid, can incur the following reaction [35]:

[Ti(OH)_3_]·Cl+ L^−^ → [Ti(OH)_3_]﹒L + Cl^−^ (3)

L^−^ is apt to form a chelate compound, which dissolves in water; this will accelerate the dissolution of passivation film (TiO_2_) on titanium. A tiny bit of the dissolved TiO_2_ causes the deficiency of the protective film, and small molecules, such as H_2_O, O_2_ and H^+^, can spread to the substrate by defects, leading to the tendency toward pitting corrosion. Therefore, the addition of lactic acid does accelerate the corrosion of titanium in artificial saliva. 

## 3. Experimental Section 

### 3.1. Preparation of the Specimens

Casting samples measuring about 10 mm × 10 mm × 1 mm were made from pure titanium with the following chemical composition (mass %): Ti ≥ 99.405, Fe ≤ 0.25, C ≤ 0.10, N ≤ 0.03, H ≤ 0.015, O ≤ 0.20. The specimen was embedded in epoxy resin disk with one flat surface exposed. The exposed surface of the specimen was abraded with #100, #320, #600, #1000, #1200 and #2000 wet silicon carbide papers to obtain a mirror-like finish, then rinsed with distilled water, degreased with acetone (CH_3_COCH_3_), sterilized with 5% glutaraldehyde solution for two hours and finally dried.

### 3.2. Medium

All tests were conducted using simulated artificial saliva with the following chemical composition (g/L): Na_2_HPO_4_ 0.26, NaCl 6.70, KSCN 0.33, KH_2_PO_4_ 0.20, NaHCO_3_ 1.50, KCl 1.20, urea 1.50, brain heart infusion broth (BHI) 37.0 and distilled water for the remainder. The pH of the medium was buffered with 0.1 M hydrochloric acid solution (HCl) at a physiological value of 6.7. Prior to the measurements, the samples were immersed in artificial saliva containing different amounts of lactic acid for different times. The corresponding concentrations of lactic acid in artificial saliva were respectively 0, 2.5, 3.2, 4.5 and 5.0 g/L.

### 3.3. pH Tests

pH tests were also performed in artificial saliva solutions containing a working electrode with and without lactic acid before electrochemical experiments. pH tests was surveyed by PHS-25 (Hongyi instrument Company, Shanghai, China) and performed in triplicate.

### 3.4. Electrochemical Examinations

The electrochemical measurements were carried out in a beaker, which is a standard three-electrode system, including a working electrode, an auxiliary electrode and a reference electrode, using a potentiostat of the PARSTAT 2263 electrochemical tester (Perkin Elmer™ company, Boston, MA, USA) interface to a personal computer. The titanium, Pt electrode and a saturated calomel electrode (SCE) were adopted as working electrodes, the auxiliary electrode and the reference electrode, respectively. All experiments were performed at a stirring speed of 150 rpm using polytetrafluoroethylene magnetic stirring in artificial saliva solutions with and without lactic acid at 37 °C.

Polarization curves were carried out after the OCP measurements, at a scan rate of 1 mV·s^−1^, from a potential of −250 mV, up to a potential of 250 mV with respect to its free corrosion potential (*E*_corr_). In order to further study the physical and chemical properties on the surface of the samples, EIS was also measured. The range of scan frequency was set from 0.1 Hz to 100 KHz. The EIS tests were repeated thrice for each specimen to guarantee the repeatability of the results.

### 3.5. Corrosion Morphology

After immersion in artificial saliva with and without lactic acid solutions for 14 days, the samples were taken out. Prior to characterizing the morphologies of corrosion, the samples were carefully washed with distilled water and then fixed by 15 min immersion in 2.5% glutaraldehyde. In order to better compare with the results of the subsequent studies in the presence of oral microorganisms, the samples were also dehydrated in a series of aqueous ethanol solutions (15%, 30%, 50%, 70%, 95% and 100%) for 15 min for sterilization; then, the surface morphology was examined by scanning electron microscopy (FEI Quanta 200, Eindhoven, The Netherlands). 

## 4. Conclusions

The corrosion behavior of titanium in artificial saliva with and without lactic acid has been investigated by means of pH tests, OCP, Tafel, EIS, SEM, *etc.* The main conclusions can be drawn as follows:

(1) The corrosion of titanium in artificial saliva will result in a slight decrease in the pH value of the solution. Additionally, the corrosion increases with increasing the immersion time.
(2) The addition of lactic acid into artificial saliva solutions can distinctly accelerate the corrosion rate, and the corrosion of titanium is aggravated with increasing the amount of lactic acid.
(3) SEM indicates that lactic acid can accelerate the pitting corrosion in artificial saliva.
(4) Lactic acid is apt to form a chelate compound ([Ti(OH)_3_]·L), which dissolves in water. The formation of [Ti(OH)_3_]·L accelerates the dissolution of passivation film (TiO_2_) on titanium, and this causes the deficiency of the protective film, leading to a tendency of pitting corrosion.
(5) The addition of lactic acid does change the mechanism, but accelerates the pitting corrosion.
